# Analysis of Collective Human Intelligence for Diagnosis of Pigmented Skin Lesions Harnessed by Gamification Via a Web-Based Training Platform: Simulation Reader Study

**DOI:** 10.2196/15597

**Published:** 2020-01-24

**Authors:** Christoph Rinner, Harald Kittler, Cliff Rosendahl, Philipp Tschandl

**Affiliations:** 1 Center for Medical Statistics, Informatics and Intelligent Systems Medical University of Vienna Vienna Austria; 2 Department of Dermatology Medical University of Vienna Vienna Austria; 3 Faculty of Medicine University of Queensland Brisbane Australia

**Keywords:** skin cancer, crowdsourcing, games, experimental, diagnosis, melanoma, nevi, skin pigmentation, basal cell carcinoma, dermatoscopy

## Abstract

**Background:**

The diagnosis of pigmented skin lesion is error prone and requires domain-specific expertise, which is not readily available in many parts of the world. Collective intelligence could potentially decrease the error rates of nonexperts.

**Objective:**

The aim of this study was to evaluate the feasibility and impact of collective intelligence for the detection of skin cancer.

**Methods:**

We created a gamified study platform on a stack of established Web technologies and presented 4216 dermatoscopic images of the most common benign and malignant pigmented skin lesions to 1245 human raters with different levels of experience. Raters were recruited via scientific meetings, mailing lists, and social media posts. Education was self-declared, and domain-specific experience was tested by screening tests. In the target test, the readers had to assign 30 dermatoscopic images to 1 of the 7 disease categories. The readers could repeat the test with different lesions at their own discretion. Collective human intelligence was achieved by sampling answers from multiple readers. The disease category with most votes was regarded as the collective vote per image.

**Results:**

We collected 111,019 single ratings, with a mean of 25.2 (SD 18.5) ratings per image. As single raters, nonexperts achieved a lower mean accuracy (58.6%) than experts (68.4%; mean difference=−9.4%; 95% CI −10.74% to −8.1%; *P*<.001). Collectives of nonexperts achieved higher accuracies than single raters, and the improvement increased with the size of the collective. A collective of 4 nonexperts surpassed single nonexperts in accuracy by 6.3% (95% CI 6.1% to 6.6%; *P*<.001). The accuracy of a collective of 8 nonexperts was 9.7% higher (95% CI 9.5% to 10.29%; *P*<.001) than that of single nonexperts, an improvement similar to single experts (*P*=.73). The sensitivity for malignant images increased for nonexperts (66.3% to 77.6%) and experts (64.6% to 79.4%) for answers given faster than the intrarater mean.

**Conclusions:**

A high number of raters can be attracted by elements of gamification and Web-based marketing via mailing lists and social media. Nonexperts increase their accuracy to expert level when acting as a collective, and faster answers correspond to higher accuracy. This information could be useful in a teledermatology setting.

## Introduction

### Background

Accurate diagnosis of pigmented skin lesions requires experience and depends on the availability of specifically trained physicians [1]. The introduction of convolutional neural networks boosted the development of decision support systems that diagnose pigmented skin lesions independent of human expertise [2]. Recently, it has been shown that computer algorithms outperform humans in the diagnosis of most types of pigmented skin lesions, including melanoma (MEL) [3]. Despite their good performance, computer algorithms are not well accepted in the medical community, probably because of the lack of interpretability of the output, suboptimal human-machine interfaces, and insufficient integration in the clinical workflow. Another limitation of algorithms is their decreased performance for out-of-distribution images [4], which impairs their generalizability. Beside machine learning algorithms, other approaches exist that are directed toward delivering expert dermatologic service for the accurate diagnosis of pigmented skin lesions. Store-and-forward telemedicine technologies are well tested for triaging [5,6], but are especially useful in regions where specialist service is not readily available [7]. Most telemedical applications use dermatoscopic images, as dermatoscopy improves the diagnosis of pigmented skin lesions in comparison with examination with the unaided eye [8].

Aside the help of computer algorithms, the use of collective intelligence could improve the accuracy of diagnosis to, or even beyond, the level of experts. Collective intelligence has emerged in the last two decades with the rise of interconnected groups of people and computers, collectively solving difficult tasks [9,10]. In radiology, for example, artificial swarm intelligence has been used to increase the diagnostic accuracy when reviewing chest x-rays for the presence of pneumonia [11].

### Objectives

To measure the effect of swarm intelligence for the diagnosis of pigmented skin lesions, we created a publicly available dataset of 10,015 dermatoscopic images and developed a Web-based study platform with elements of gamification. We also aimed at providing a publicly available benchmark dataset with human diagnoses and corresponding metadata that will be helpful for developing and testing future machine learning algorithms.

The aim of this study was to find out whether a collective of nonexperts can reach expert-level (ie, being frequently consulted by experts and advanced users) accuracy in diagnosing skin cancer on dermatoscopic images and to find out the collective size needed for this.

## Methods

### Web-Based Training Platform

The Web-based platform DermaChallenge [12], which was developed at the Medical University of Vienna, is an interactive training platform to educate dermatologists and other physicians interested in dermatoscopy via gamification and individual feedback. The platform is split into the back end and the front end, and both are deployed on a stack of well-known Web technologies (Linux, Apache, MySQL, and PHP) at the Medical University of Vienna. The back end is programmed using Laravel (version 5.5) [13] and offers a Representational State Transfer interface to load and persist data as well as JavaScript Object Notation Web tokens to authenticate participants. To protect participants’ data, the Transport Layer Security and Secure Sockets Layer protocol are used to encrypt all communications. The front end is a React (version 16) [14] app optimized for mobile devices (mobile phones and tablets) but can also be used on any other platform via a JavaScript-enabled Web browser. The user interface is based on Semantic UI React [15] components and Redux [16] to manage the state of the application. Before public deployment, 5 users tested the platform.

To participate in the study, participants had to register with a username, a valid email address, and a password. In addition, we asked participants their age (age groups spanning 10 years), gender, country, profession, and years of experience in dermatoscopy. Ordered options for the last item were (1) less than 1 year, (2) opportunistic use for more than 1 year, (3) regular use for 1 to 5 years, (4) regular use for more than 5 years, or (5) more than 10 years of experience. Groups 1 to 4 were regarded as *nonexperts* and group 5 as *experts*. The training platform was publicly available; to start playing, only registration had to be completed and the email address had to be verified.

For gamification, the training platform is structured into stepwise levels with different tasks of varying degrees of difficulty. As the platform is available to any user with a valid email address, we needed to verify plausibility of self-declared experience status. For this, the first 3 levels were *screening levels* and comprised simple domain-specific tasks (assign 1 of the 7 possible diagnoses to 10 cases, separate MELs from non-MELs, and separate seborrheic keratoses from other lesions) to introduce the platform and to assess the basic skills of the participants (see Tschandl et al [3]). The target level (level 4) was available only after the completion of 1 round in each screening level (levels 1-3). In the target level, 30 dermatoscopic images were presented to the participants. The images in this level were taken from a master dataset of 10,015 dermatoscopic images (the Human Against Machine with 10000 training images (HAM10000) dataset [17], publicly available at The Harvard Dataverse [18] and the ISIC-Archive [19]), where all malignant diagnoses were verified via histopathology, and ground truth for benign cases was distributed in a similar fashion to the source dataset. The task of the raters in this level was to select the correct diagnosis out of 7 predefined categories: (1) actinic keratosis/intraepithelial carcinoma (AKIEC), (2) basal cell carcinoma (BCC), (3) seborrheic keratosis/solar lentigo/lichen planus–like keratosis (*benign keratinocytic lesions*, BKL), (4) dermatofibroma (DF), (5) MEL, (6) nevus (NV), and (7) vascular lesions (VASC). In clinical practice, more than 95% of pigmented skin lesions will fall into 1 of the 7 categories [8]. The batches of 30 images per round had a predefined composition of diseases (3×AKIEC, 4×BCC, 4×BKL, 3×DF, 5×MEL, 9×NV, and 2×VASC). Cases were selected randomly from each disease category according to this blueprint. To ensure a balanced distribution of NV, this category was stratified into 3 groups according to ground truth as published with the HAM10000 dataset (histopathology, follow-up with digital dermatoscopy, and expert consensus [17]). Each batch of NV included 3 cases of each category. In the analysis of this paper, only data from these 4 levels are used.

Raters were allowed to play more than 1 round per level. When a round was completed, raters were able to see their scoring rank in an *all-time* or *current month* leaderboard for each level and could compare their accuracy with others. To avoid cheating, each image was shown for a maximum of 25 seconds. After 20 seconds, raters received a warning that the system will continue to the next case in 5 seconds. If no answer was given after the time expired, the answer was counted as invalid and not included in this study. After completion of a full round, the participants were able to review their diagnoses and compare them with the correct diagnosis. In the background, the platform stored the selected diagnosis, the current level and round, the answering time, and the screen resolution.

### Recruitment, Registration, and Engagement

We used mailing lists, social media posts, and talks at scientific conferences to recruit participants. To compare recruitment strategies, we continuously monitored the number of new registrations and related them to specific recruitment events, if they occurred within 4 days after the event. To analyze registration and dropout, we categorized participants into (1) registered but not verified by email; (2) registered and verified but did not complete any level; (3) registered, verified, played, and completed 1 of the screening levels at least once; and (4) registered, verified, played, and completed all screening levels and the target level at least once. We analyzed engagement with the unbounded retention measure used in game analytics [20] by calculating how many participants returned and played at least one level between 0 and 100 days after their registration. The device types were identified using the free Web analytics software Matomo [21].

### Accuracy and Collective Intelligence

We calculated baseline measures of accuracy (ie, correct specific prediction of a disease category, not just malignancy) for each dermatoscopic image and per disease category for single raters. To calculate the measures of accuracy for collective intelligence, we applied bootstrapping (random sampling with replacement), simulating multiple second opinions. We let the size of the collectives range from 3 to 8. Dermatoscopic images for which the number of raters was lower than the size of the virtual collective were excluded. The disease category with most votes (ie, first-past-the-post voting) was regarded as the collective vote per dermatoscopic image; ties were broken at random. This procedure was repeated for answers of nonexperts as well as for the answers with high and low confidence.

We used the answering time as a surrogate measure for the level of confidence for each image. To allow an unbiased comparison, we calculated the mean answering time for every rater individually. Furthermore, if a rater needed more time than the mean individual answering time for the disease category, the level of confidence for a given answer was regarded as low. If, on the other hand, the answering time was lesser than or equal to the individual mean of this specific rater, the level of confidence was regarded as high. The confidence *all* represents all answers, including low- and high-confidence answers.

The primary outcome metric is the mean accuracy, which we defined as the arithmetic mean of accuracies of every image within a rater group. All calculated accuracies per image were compared pairwise with the baseline accuracy of nonexperts. Point estimates for the difference in accuracy, confidence intervals, and *P* values were only calculated for pairwise overlapping images among groups. Sensitivity, specificity, and positive and negative predictive values were used when the number of analyzed classes was binary, as for the case of malignancy. As part of this study, the diagnostic groups *AKIEC*, *BCC*, and *MEL* are regarded as *malignant* and all others (*BKL*, *DF*, *NV*, and *VASC*) as *benign*, ie, a prediction of *BCC* is counted as correct if *MEL* was the ground truth.

As some raters took the target level significantly more often than others, we restricted the number of rounds per rater to 30 to prevent bias. If the rounds were not completed, we included the answers only if more than 50% of cases (ie, 15 dermatoscopic images) per round were rated.

### Statistical Methods and Ethics

Classic measures of diagnostic values (sensitivities, specificities, and predictive values) were calculated per rater group and according to standard formulas [22] for detecting a malignant skin lesion, where prediction of any type of malignant disease category (ie, AKIEC, BCC, or MEL) was considered a correct prediction for any malignant image.

Descriptive continuous values are presented as mean with standard deviation; estimates are provided with 95% confidence intervals. We used paired *t* tests for comparing the difference in correct answers for images between single raters and bootstrapped collective intelligence procedures. All *P* values are reported corrected for multiple testing (Bonferroni-Holm [23]) unless otherwise specified, and a 2-sided *P* value <.05 was regarded as statistically significant. Calculations and plotting were performed using R version 3.4.0 [22] and ggplot2 [24].

The study was approved by the ethics review boards of the University of Queensland (protocol number 2017001223) and the Medical University of Vienna (protocol number 1804/2017). During registration, human raters provided written consent to allow analyzing anonymized ratings. A total of 4 participants demanded all their data to be deleted; therefore, their ratings are not included in this study.

## Results

### Registration, Recruitment, and Engagement

Of the 2497 individuals (1538/2497, 61.59% female) who registered between June 15, 2018, and June 14, 2019, 44.09% (1101/2497) were board-certified dermatologists, 25.55% (638/2497) were dermatology residents, and 16.58% (414/2497) were general practitioners. In the 365 days, the survey page was visited 21,948 times. The raters came from 5 continents (Africa, n=112; Asia, n=204; Europe, n=1260; Americas, n=594; and Australia/Oceania, n=327).

The raters used mobile phones in 56.80% (13,042/22,961), a desktop computer in 30.80% (7061/22,925), and a tablet in 6.70% (1546/23,074) of visits to the survey page. The mean time spent on the site per visit was 4 min 37 seconds (SD 3 min 2 seconds). Of the 2497 registered raters, 367 (14.69%) dropped out before playing at least one level, 1330 (53.26%) completed the screening levels and started playing the target level, and 1245 (49.85%) completed the target level at least once. The distribution of age, gender, continent of origin, and experience was similar among registered raters who finished the screening tests and played the target level and those who dropped out. Peaks of registrations could be attributed to specific recruitment events. Most participants were recruited from social media (701/2497, 28.07%) or through mailing lists (732/2497, 29.31%). Only 1.96% (49/2497) of the participants were recruited from scientific meetings; the remaining 40.64% (1015/2497) could not be attributed to a specific event. The highest number of participants recruited per day was 676, after a social media campaign. Without any social media marketing, the number of visitors spanned from 15 to 40 visitors per day (at the time of submission). Participants with less than 1 year of experience had the lowest 30-day unbounded retention rate (21.9%), and participants with less than 3 years of experience had the highest 30-day unbounded retention rate (33.7%).

In the target level, we collected 111,019 single ratings, with a mean of 25.2 (SD 18.5) ratings per image. Only the 4216 images with 8 or more ratings were included in this analysis (AKIEC, n=327; BCC, n=514; BKL, n=1099; DF, n=115; MEL, n=1113; NV, n=907; and VASC, n=142). At the nonexpert level, data of 1208 participants, 4216 different images, 4102 rounds, and 101,271 ratings were included. At the expert level, data of 37 participants, 2609 different images, 193 rounds, and 4762 ratings were included.

### Collective Human Intelligence

Table 1 shows the mean accuracies achieved by single experts, single nonexperts, and collectives of nonexperts with different group sizes. We did not calculate the accuracies for the collective intelligence of experts as individual images were not seen by a sufficient number of experts.

**Table 1:** Comparison of mean accuracy of single nonexperts to mean accuracy of different collective sizes and confidence levels. *P* values denote paired t test comparing the number of correct specific diagnoses per overlapping dermatoscopic image.

Experience	Collective size	Confidence^a^	Mean accuracy, %	Mean difference (95% CI)	*P* value
Nonexperts	—^b^	All	58.60	Reference	Reference
Nonexperts	4	All	64.93	6.33 (6.09 to 6.57)	<.001
Nonexperts	8	All	68.51	9.91 (9.52 to 10.29)	<.001
Nonexperts	—	Low	51.90	−6.20 (−6.77 to −5.64)	<.001
Nonexperts	4	Low	56.01	−2.10 (−2.72 to −1.47)	<.001
Nonexperts	8	Low	59.27	1.16 (0.45 to 1.88)	.007
Nonexperts	—	High	61.40	2.77 (2.44 to 3.09)	<.001
Nonexperts	4	High	65.85	7.22 (6.77 to 7.66)	<.001
Nonexperts	8	High	68.40	9.77 (9.21 to 10.32)	<.001
Experts	—	All	68.36	9.43 (8.11 to 10.74)	<.001
Experts	—	Low	55.61	4.67 (2.27 to 7.06)	<.001
Experts	—	High	74.08	11.91 (10.43 to 13.38)	<.001

^a^Confidence groups denote whether all answers of raters were measured (All) or only answers given with low or high confidence.

^b^No collective size.

Nonexperts with low confidence had the lowest mean accuracy (51.9%, SD 28.9), whereas confident experts had the highest mean accuracy (74.1%, SD 41.7). A *collective of 8 confident nonexperts* had a similar mean accuracy difference to *single nonexperts* as *single experts* (+9.77 vs +9.43; *P*=.73).

**Figure 1:**
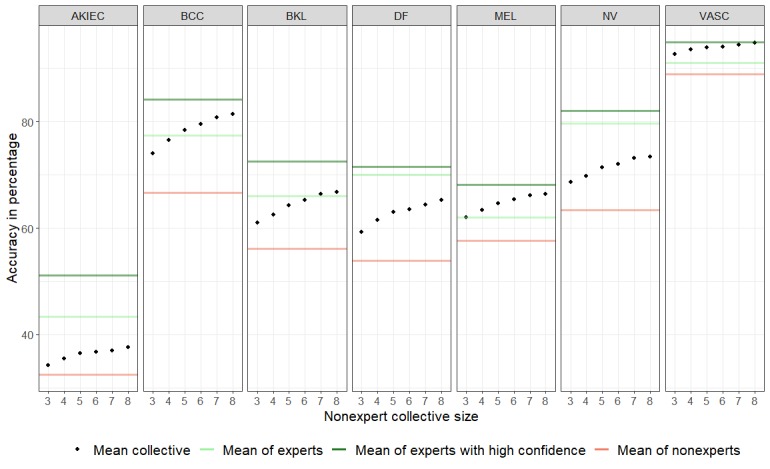
Mean accuracy (dots) per disease category of nonexpert collectives (ranging from 3 to 8) compared with the mean sensitivity of single experts, single experts with high confidence, and single nonexperts. AKIEC: actinic keratosis/intraepithelial carcinoma; BCC: basal cell carcinoma; BKL: benign keratinocytic lesions; DF: dermatofibroma; MEL: melanoma; NV: nevus; VASC: vascular lesions.

Figure 1 shows the mean sensitivity for each of the 7 disease categories for collectives ranging from 3 to 8 compared with the mean sensitivity of single experts and nonexperts. For all disease categories, the mean sensitivity improved with increasing size of the collective. The mean sensitivity for the disease categories VASC and MEL for collectives of 3 raters was already higher than that for single experts. For BCC and BKL, a collective of 5 and 7, respectively, was needed to surpass the mean accuracy of single experts.

The mean time to answer a case was below 5 seconds for both nonexperts (4.7 seconds, SD 4.05) and experts (3.9 seconds, SD 3.47), below 3 seconds in case of high confidence (2.8 seconds, SD 1.75 and 2.3 seconds, SD 1.29, respectively), and above 7 seconds in case of low confidence (7.0 seconds, SD 4.74 and 7.0 seconds, SD 4.22, respectively). The sensitivity for malignant cases increased for both nonexperts and experts in the presence of high confidence compared with low confidence (low vs high nonexperts: low 66.3% vs high 77.6% and experts: low 64.6% vs high 79.4%; see Table 2).

**Table 2:** Diagnostic values measuring detection of malignant skin lesions for different confidence levels.

Experience	Collective size	Confidence^a^	Sensitivity, % (95% CI)	Specificity, % (95% CI)	Positive predictive values, % (95% CI)	Negative predictive values, % (95% CI)
Nonexpert	—^b^	All	73.1 (72.7 to 73.5)	77.4 (77.0 to 77.7)	75.1 (74.7 to 75.5)	75.5 (75.1 to 75.9)
Nonexpert	4	All	74.6 (73.9 to 75.2)	78.0 (77.5 to 78.5)	74.5 (73.9 to 75.2)	78.0 (77.5 to 78.6)
Nonexpert	8	All	76.9 (76.3 to 77.5)	80.1 (79.6 to 80.6)	77.0 (76.4 to 77.6)	80.1 (79.5 to 80.6)
Nonexpert	—	Low	66.3 (65.6 to 67.0)	69.7 (69.1 to 70.4)	69.4 (68.7 to 70.1)	66.7 (66.0 to 67.3)
Nonexpert	4	Low	68.7 (68.0 to 69.4)	74.6 (74.0 to 75.2)	70.3 (69.7 to 71.0)	73.1 (72.5 to 73.7)
Nonexpert	8	Low	70.9 (70.3 to 71.6)	76.5 (75.9 to 77.0)	72.5 (71.9 to 73.2)	75.0 (74.4 to 75.6)
Nonexpert	—	High	77.6 (77.1 to 78.0)	81.6 (81.2 to 82.0)	78.7 (78.3 to 79.2)	80.5 (80.1 to 81.0)
Nonexpert	4	High	76.9 (76.3 to 77.5)	77.6 (77.0 to 78.1)	74.8 (74.2 to 75.4)	79.5 (79.0 to 80.0)
Nonexpert	8	High	78.3 (77.7 to 78.8)	79.0 (78.4 to 79.5)	76.3 (75.7 to 76.9)	80.8 (80.2 to 81.3)
Expert	—	All	74.0 (72.2 to 75.8)	85.8 (84.4 to 87.2)	83.1 (81.4 to 84.7)	77.8 (76.2 to 79.4)
Expert	—	Low	64.6 (61.3 to 67.8)	77.4 (74.3 to 80.3)	75.6 (72.2 to 78.7)	66.9 (63.7 to 70.0)
Expert	—	High	79.4 (77.2 to 81.4)	89.7 (88.2 to 91.1)	87.1 (85.2 to 88.9)	83.3 (81.5 to 85.0)

^a^Confidence groups denote whether all answers of raters were measured (All) or only answers given with low or high confidence.

^b^No collective size.

## Discussion

### Principal Findings

In this study, we showed that collective human intelligence increases the accuracy of nonexperts for the diagnosis of pigmented skin lesions. As collectives, nonexperts reached expert-level accuracies. Although experts were significantly more accurate than nonexperts in general, this difference vanished when average experts were compared with collectives of 8 nonexperts (Table 1). For specific diagnoses, a group of 3 to 8 nonexperts surpassed the sensitivity of the average expert (Figure 1). Potentially, this information could be used for telemedical applications, where a small number of overburdened experts evaluate the majority of referred cases. Small and dynamic groups of physicians, regardless of their expertise, could be used as an alternative to experts. With this strategy, it is possible to recruit raters from a large pool of physicians.

We also found that not all ratings from nonexperts were equally helpful. The ratings given with lower confidence, defined as slow answers in comparison with the mean answer time of a rater, did not increase the accuracy of the collectives of nonexperts. The ratings given with lower confidence even reduced the mean accuracy of small collectives (Table 1). As a consequence, such nonconfident answers should probably be omitted in telemedical applications, which would demand tracking of the time a rater takes to reach a decision. A limitation is that our study rating platform deviated from the real telemedicine platforms as the raters had a maximum of 25 seconds to answer, no option for explanation, and no liability for potential misdiagnoses. Our results with regard to answer time are in line with previous research on a clinical practice colloquially called “if in doubt—cut it out.” Moscarella et al [25] suggested to excise or biopsy lesions for which a specific benign diagnosis cannot be made with confidence. From the data presented herein, the decision boundary on whether there is any *doubt* may be at about 3 seconds. As we also show markedly decreased specificity in low-confidence ratings, a mandatory biopsy in those situations may increase unnecessary interventions; instead, alternative assessments may be sought, such as comparison with other lesions, follow-up imaging, or reflectance confocal microscopy.

### Strengths and Limitations

In practice, collective intelligence models, as simulated herein, could be harnessed in different ways to obtain second opinions in difficult cases. Although our method is mainly suitable for store-and-forward approaches, one could also think of real-time simultaneous interaction among readers to possibly further increase accuracy [11]. However, such an approach of swarm intelligence would require participants to be engaged continuously throughout the decision process, evaluating and reevaluating their answer depending on the real-time input of the other participants. Such an interactive swarm could be considered closer to a live discussion with colleagues, whereas the collectives simulated in this study are closer to a classic store-and-forward telemedical approach. Our collectives currently have unclear liability in case of a misdiagnosis: would each member of a collective, the provider of a platform offering collectives, or solely the treating physician be accountable for a misdiagnosis? This conundrum will be equally interesting for computer-aided diagnostics as it is conceivable that one or more members of such collectives could be replaced by a computer algorithm behind the scenes.

Our results are promising with regard to the detection of malignant skin lesions. A collective of 8 confident raters was able to raise single nonexperts’ sensitivity from 73.1% to 78.3% and specificity from 77.4% to 79.9%. Interestingly, although the mean specific accuracy of 8 confident nonexperts was at the level of experts (+0.34% difference), their operating point regarding sensitivity and specificity was more in favor of sensitivity. Therefore, such a nonexpert collective would detect more malignant skin lesions at the cost of more interventions. This, however, may be mitigated by a second line of assessments.

The diagnostic accuracy alone will not be the only consideration in a potential implementation of collective ratings in practice. Although the availability of nonexperts is higher than that of experts, the more the nonexperts involved, the higher the costs and the longer it will take to get a collective vote. With regard to the optimal number of nonexperts, the benefits, such as gain in accuracy for each additional rater, will have to be weighed against these costs. For example, 4 confident nonexperts increase the sensitivity substantially in comparison with a single unconfident nonexpert (from 66.3% to 76.9%), but the additional gain achieved by 8 nonexperts is only marginal (78.3%).

In this study, only dermatoscopic images of pigmented skin lesions were included; however, we estimate that similar improvements are possible with nonpigmented tumors and inflammatory diseases [26], which are more challenging in clinical practice, as shown in previous experiments [27]. A remaining obstacle for application in these areas is a much greater number of possible diagnoses.

We also demonstrated that a high number of raters could be attracted by online marketing and by including elements of gamification. Readers with little experience had a lower unbounded retention rate, which can probably be enhanced by adding additional elements of gamification such as avatars, progress bars (ie, Zeigarnik effect [28]), and better individual metrics or adjusting the learning difficulty level to match the participants’ level.

## Abbreviations

AKIEC: actinic keratosis/intraepithelial carcinoma
BCC: basal cell carcinoma
BKL: benign keratinocytic lesions
DF: dermatofibroma
HAM10000: Human Against Machine with 10000 training images
MEL: melanoma
NV: nevus
VASC: vascular lesions
